# Effect of Yangling inclined trellis tree shape on light interception efficiency, fruit quality, and yield of sweet cherry *cv:* ‘Jimei’

**DOI:** 10.1371/journal.pone.0317101

**Published:** 2025-03-05

**Authors:** Juan Zhang, Wei Xu, Zhiqiang Dou, Liuyi Pan, Tian Wan, Feng An, Zhifang Yang, Yuliang Cai

**Affiliations:** 1 College of Horticulture, Northwest A&F University, Yangling, Shaanxi, China; 2 College of Horticulture and Forestry, Tarim University, Alar, Xinjiang, China; 3 National and local joint Engineering Laboratory of High-Efficiency and Good Quality Cultivation and Deep-processing Technology of Characteristic Fruit Trees in Southern Xinjiang, Alar, Xinjiang, China; 4 Xinjiang Production & Construction Crops Key Laboratory of Facility Agriculture, Tarim University, Alar, Xinjiang, China; 5 Qishan County Forestry Workstation, Baoji, Shaanxi, China; Nuclear Science and Technology Research Institute, IRANISLAMIC REPUBLIC OF

## Abstract

Different tree shapes (TSs) directly influence canopy structure, light interception, and photosynthetic activity, impacting fruit quality and yield. This study investigated the effects of light interception efficiency (LIE) on the quality and yield of “Jimei” sweet cherry fruits by comparing two tree shapes: the Yangling inclined trellis arm TS (YLL-TS) and the Super slender spindle TS (SSS-TS). Using three-dimensional digitization techniques, we analyzed the growth relationships between branches and leaves, constructed virtual canopy models, and examined branch composition, leaf area, and spatial distribution. The present study indicated varying correlation coefficients for growth relationships between branches and leaves in the two TSs. For YLL-TS, the smallest correlation coefficient was between the length of nutrient-bearing branches and leaf petiole length (r² =  0.206), while the largest was between the length of short fruit-bearing branches and the number of branches and leaves (r² =  0.851). For SSS-TS, the smallest was between the length of medium fruit-bearing branches and leaf petiole length (r² =  0.211), and the largest was between the length of nutrient-bearing branches and leaf area (r² =  0.827). The LIE for YLL-TS (0.53 STAR value) was significantly higher than SSS-TS (0.20 STAR value). Although YLL-TS had fewer branches and leaf area, it showed increases in LIE by 48%, 42%, and 27% for overall canopy, fruit-bearing branches, and nutrient-bearing branches, respectively. The photosynthetic parameters (Pn, Tr, Gs, and Ci) were higher in SSS-TS. YLL-TS exhibited a higher economic yield (4.075 kg/ m^2^) and is more suitable for dense planting, facilitating widespread cultivation.

## 1. Introduction

To survive, reproduce, and coexist under various environmental and ecological conditions, plants have evolved a diverse array of structure [1,2]. Plant structure is crucial for resource use efficiency, as it forms the core of the fundamental balance between carbon acquisition and water utilization, influencing leaf area distribution, light interception, water transport and loss, as well as carbon assimilation and allocation [3]. The importance of light for crop production is indisputable. Acquiring light energy is essential for plant survival and growth, as it provides the necessary energy [4]. Light interception within the canopy of fruit trees affects leaf energy balance and further influences transpiration. Therefore, increased light interception can lead to higher transpiration demand, resulting in greater soil moisture depletion and severity of water deficit [3]. The yield and quality of fruit depend on the light microclimate within the trees. Fruit yield is related to total light interception in healthy and well-watered trees [5]. Canopy light interception determines the amount of energy available to crops, which is crucial for growth and yield modelling, and may significantly increase the predictive uncertainty of crop growth models (CGMs) [6].

To achieve early and increased fruiting and yield in orchards, pruning is a common approach in fruit tree production. Differences in canopy morphology resulting from pruning can significantly alter light transmission to various regions of the canopy, while fruit quality and yield vary with changes in canopy position and are closely related to light transmission rates [7]. Different tree shapes (TSs) not only affect the overall tree structure but also influence light utilization efficiency and photosynthetic efficiency. They also impact current-year tree vigor, yield, fruit quality, and orchard management efficiency, thereby affecting the quantity and quality of fruit bud differentiation, as well as the yield and quality in the following year. Canopy size, structure, branch distribution, and quantity are fundamental elements of TSs [8]. Proper pruning adjusts canopy size and branch quantity, improving ventilation and light transmission conditions. Effective light utilization is the foundation of fruit tree yield formation. Different TSs aim to balance light interception, yield quality, and the nutritional and reproductive growth of trees.

Sweet cherry trees are heliophilic. During the fruiting period, mature trees with vigorous branch growth may experience insufficient light within the canopy, leading to internal canopy shading and outward shifting of fruit-bearing positions [9]. Additionally, improper pruning and extensive management can cause decreased yield and quality. In China, research on the canopy structure and light interception efficiency (LIE) of sweet cherry trees is limited. Previous reports mainly focus on the distribution of effective light radiation and basic photosynthetic characteristic parameters, lacking research on overall canopy structure and light interception capacity [10,11].

In this study, we used the sweet cherry cultivar ‘Jimei’ with spindle bush and YLL-TS as experimental material. Employing three-dimensional digitization technology and virtual plant technology, we accurately constructed canopy models of different TSs and evaluated the LIE, fruit quality, and yield of sweet cherry trees with different TSs. Our study provides a theoretical basis for shaping and pruning cherry trees and addresses issues such as canopy closure. This research aims to achieve high-quality and high-yield cherry orchards through relevant data analyses.

## 2. Materials and methods

### 2.1 Plant material and growth conditions

The experiment was conducted at the Cherry and Apple Experimental Station of Northwest A&F University in Tongchuan from 2020 to 2021 (located at 108°49’ east longitude and 34°51’ north latitude, in mountainous terrain with an elevation ranging from 822.2 to 829.1 MSL). The experimental site has a temperate continental climate, with an average annual sunshine duration of 2356.6 hours, average annual precipitation of 554.5 mm, and average annual temperature ranging from 8.4 °C to 12.3 °C. The lowest temperature recorded annually is -12 °C, with a frost-free period of 210-220 days. The soil at the experimental site is loam, with a pH of approximately 7.8.

The experimental material used was ‘Jimei’ (rootstock: ‘Mahali CDR-1’), which was 7 years old and pruned to standard tree vigor equivalency. The experimental trees were of the YLL-TS, while the SSS-TS was used as the control. There were 3 trees of each TS, and the spacing between rows for both shapes were 2m ×  4.5m.

### 2.2 Collection of basic tree architecture

In 2020, basic architecture of the ultra-long spindle of tress and the YLL-TSs was measured using a tape measure. The measured parameters included tree height, crown diameter, trunk circumference, trunk height (height from the ground to the first main branch), number of main branches, and main branch angles (angle between the central trunk and the main branches).

### 2.3 Digitization of the tree canopy

**Fig 1:**
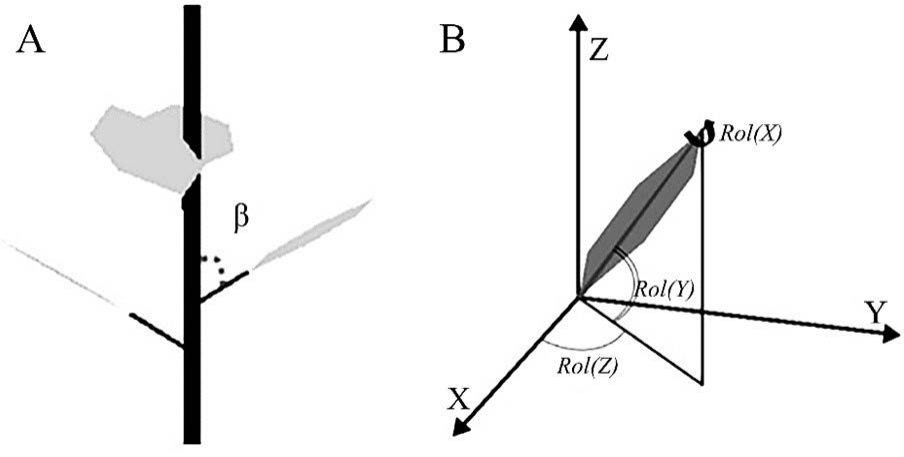
Images of representative angle between petiole and shoot axis (A) and leaf Euler angles (B).

The digitization of the cherry tree structures in both slender spindle and YLL-TS was conducted using a three-dimensional digitizer. The digitization process involved the following steps: First, a coordinate system was established using the horizontal plane as a reference, with each experimental tree having its own coordinate system (utilizing a right-handed Cartesian coordinate system, where the X-axis points towards geographic north, the Y-axis points towards geographic east, and the Z-axis points vertically downwards). Then, a pen-shaped sensor (Stylus, Polhemus Inc., Rochester, VT, U.S.A.) was used within the magnetic field generated by the transmitter to determine the spatial coordinates of the desired points, and the data was recorded in real-time using the PiafDigit software to complete the digitization of the tree structure [12]. It is crucial to note during the digitization process that the operation of the three-dimensional digitizer relies on magnetic principles, and thus, significant electromagnetic fields may interfere with the instrument’s operation. Therefore, external factors influencing the digitization process need to be avoided. Additionally, weather conditions can also affect the digitization process, so the process was conducted during low or no wind conditions to minimize the impact of wind speed on the spatial positioning of the tree structure (Fig 1).

The digitization of branches and leaves constitutes two parts of the tree digitization process. In this experiment, the digitization was conducted approximately one month before the cherry fruit ripened. The digitization of branches involved digitizing all new shoots and one-year-old branches, with the spatial coordinates of the base and tip of each shoot determined using the pen-shaped sensor. Based on the spatial distribution characteristics of leaves on different types of branches, the branches were classified into the following categories: long fruit shoots (LS) (≥25 cm), middle fruit shoots (MS) (10-25 cm), short fruit shoots (SS) (5-10 cm), spurs (SP) (≤2 cm), as well as vegetative long shoots (VL) (>30 cm) and vegetative short shoots (VS) (0-30 cm) [13].

Further the digitization of branches, to obtain characteristics such as the spatial distribution and angles of leaves, 15-20 branches of each type were randomly selected from each treatment, and each leaf on the branches was digitized. An RX1-D sensor was placed at the intersection of the petiole and leaf to ensure that the sensor was parallel to the leaf, allowing for the measurement of leaf Euler angles (azimuth angle, elevation angle, and roll angle) and the angle between the petiole and the branch. During the digitization of branches and leaves, the Piaf Digit software was used to record the coordinate and angle values in real-time.

After the digitization of the tree structures, the allometric growth was determined to draw the relationship between branches and leaves. In this experiment, 15 branches of each branch type were selected and brought back to the laboratory for statistical analysis of branch length (SL) and the number of leaves on each branch (LN). Subsequently, morphological parameters of different leaf types on different TSs were measured. These parameters included petiole length (PL), leaf length (LL), and leaf width (LW). Additionally, a leaf area meter (Yaxin-1242) was used to calculate the leaf area of individual leaves (LA), and the total leaf area of individual branches (SLA) was calculated [14], aiming to establish the allometric growth relationship between branches and leaves to provide data support for the subsequent reconstruction of the three-dimensional crown structure.


$$L\left(shoot\right)=\sqrt{{\left(X_{T}-X_{B}\right)}^{2}+{\left(Y_{T}-Y_{B}\right)}^{2}+{\left(Z_{T}-Z_{B}\right)}^{2}}$$ (1)

Where, X_B_, Y_B_ and Z_B_ are the base special coordinates of the branch, and X_T_, Y_T_ and Z_T_ are the vertex special coordinates of the branch

### 2.4 Simulated reconstruction of the canopy

**Fig 2:**
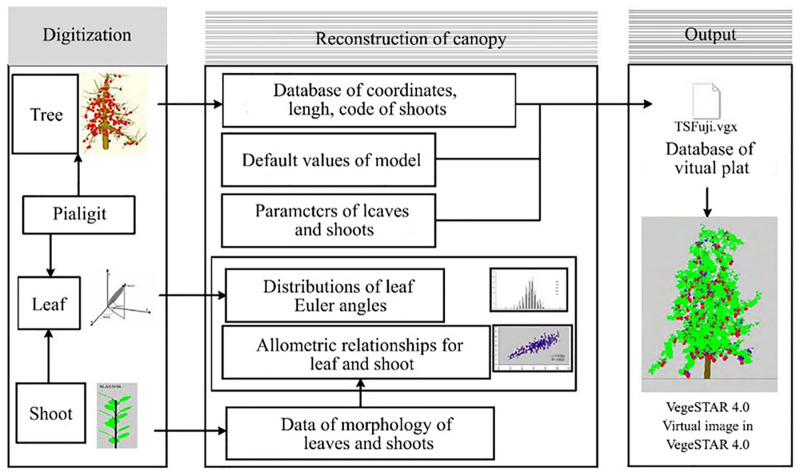
Structural view of model.

The three-dimensional reconstruction of the canopy was measured based on the digital data of branches and leaves. By utilizing the spatial coordinates of the base and apex of branches obtained from branch digitalization and combining them with Shapeula [6], the simulated length of branches is derived. To obtain the distribution of leaves on each type of branch and the branch leaf area, the allometric growth relationship between branch length and leaf number, as well as leaf area, is utilized. For simulating individual leaves on branches, the aforementioned allometric growth relationship between branch length and leaves is coupled. Finally, based on the rules of simulation reconstruction, the allometric growth relationship between branches and leaves, and relevant algorithms, a database file of various branches and leaves of the cherry tree is generated using the obtained spatial coordinates of all branches. This database file is used to generate a three-dimensional simulated image of the cherry tree canopy in VegeSTAR 4.0. The process of digitizing the canopy and reconstructing the canopy is depicted in Fig 2 [14].

Using the VegeSTAR 4.0 software, simulated images of the cherry tree canopy are generated, and the LIE of the cherry tree canopy is evaluated. In the VegeSTAR 4.0 software, the geographical location of the experimental site is set, and sunlight at a specific moment at the local site is simulated. The portion of the canopy where leaves receive direct sunlight is referred to as the Projected Leaf Area (PLA). The total leaf area of the canopy (Total Leaf Area, TLA) is calculated in the software. The ratio of PLA to TLA, termed as STAR (Silhouette to Total Leaf Area Ratio), is used as the basis for calculating the LIE of the canopy [15]. Additionally, by setting colors for different types of branches, the STAR values for each type of branch are calculated. During the calculation of light interception, the VegeSTAR 4.0 software simulates a Turtle Sky, which is divided into 46 equally sized sky directions [5]. Finally, the STAR values obtained from the 46 sky directions of the Turtle Sky are weighted and summed up to derive the overall STAR value. Furthermore, based on the digitally collected data of branches and leaves, an assessment of the canopy structure for both types of cherry TSs are conducted, including metrics such as branch quantity, leaf number, and leaf area.


STAR=PLATLA (2)

Where, PLA is Projected Leaf Area; TLA is Total Leaf Area

### 2.5 Validation of simulated canopy accuracy

After completing the reconstruction of the sweet cherry tree canopy model, the accuracy of the reconstruction is validated. The validation of the accuracy of the three-dimensional model of the sweet cherry canopy primarily involves analyzing the simulated leaf area and the measured leaf area. The accuracy of leaf area simulation is verified using regression root mean square error (RMSE) [6] and relative error (RE) [6], serving as criteria for assessing the precision of the simulated values.


$$RMSE=\sqrt{\frac{\sum_{i=1}^{n}{\left(S_{A}-S_{B}\right)}^{2}}{n}}$$ (3)

RE=RMSE1n∑i=1nSA (4)

Where, S_A_ is simulated leaf area and S_B_ measured leaf area.

### 2.6 Measurement of leaf photosynthetic properties

Between May and June 2021, on sunny days, the photosynthetic characteristics parameters of the leaves of two types of sweet cherry trees were measured using a Li-6800 photosynthesis analyzer. The measurement was conducted by selecting the sixth leaf from the top downwards on the terminal shoots or one-year-old branches, with five leaves chosen from each selected position. The final results were obtained by averaging the measurements. The measurement locations were divided vertically into three parts based on a standard of 1 meter: upper part (above 2 meters from the ground), middle part (between 1 meter and 2 meters from the ground), and lower part (from the ground to 1 meter above). The measurement method for the photosynthetic characteristics of the leaves at each location followed the procedure.

### 2.7 Determination of fruit yield

One week before the harvest period in June 2021, fruit picking was conducted, and 30 fruits were collected from each tree of different shapes. The single fruit weight was measured using an electronic scale manufactured by ATAGO. For each experimental tree, the number of fruits per plant was counted for both TSs. By combining the single fruit weight of each TS, the fruit yield per plant for both TSs was estimated. The estimation of the total fruit yield per acre for both TSs was calculated based on the different planting densities and the number of plants per acre for each TS.

### 2.8 Calculation of orchard productivity

Based on the actual statistics of the individual plant yield, main stem cross-sectional area, and the total leaf area obtained from the three-dimensional digitization of the canopy for both TSs, the unit stem cross-sectional area yield (kg/cm^2^) and unit leaf area yield (kg/m^2^) are calculated. The shapeulas are as follows:


$$\text{Unit stem cross}-\text{sectional area yield}=\frac{\text{Individual plant yield }}{\text{Main stem cross}-\text{sectional area}}$$ (5)

$$\text{Unit leaf area yield}=\frac{\text{Individual plant yield }}{\text{Total leaf area of the tree }}$$ (6)

Here, the main stem cross-sectional area is estimated based on the actual measurement of the circumference of the sweet cherry tree trunk.

### 2.9 Data analysis

The experimental data can be processed and computed using SPSS 8.0 and Excel 2010, while data visualization can be done using Excel 2010 and Origin 2019 software. For conducting significance analysis on the data, SPSS 17.0 can be employed for statistical tests such as t-tests, analysis of variance (ANOVA), etc., to assess whether differences between different variables are statistically significant.

## 3. Results

### 3.1 Analysis of basic tree data and branching percentages

The study observed that the average tree height of YLL-TS and SSS-TS were 362.33 cm and 382.67 cm, respectively, with no significant difference between them (Table 1). However, significant differences were found in the circumference and east-west canopy diameter compared to the control. The average circumference of YLL-TS was 40.63 cm, while the circumference of SSS-TS was 1.3 times that of YLL-TS. Both the east-west and north-south crown diameters of SSS-TS were greater than those of YLL-TS, exceeding 220 cm. Both TSs had more than 35 main branches with angles greater than 90°, though YLL-TS had fewer main branches and smaller branch angles than SSS-TS, with no significant difference between the two (Table 1).

**Table 1:** Statistics on the basic situation of the tree.

TS	Height of tree/cm	Height under first branch/cm	Trunk perimeter/cm	Crown width/cm		Number of main branch	Angle of main branch/°
				E-W	S-N		
SSS-TS(CK)	382.67a	82.00a	54.83a	314.13a	225.64a	41.67a	106.20a
YLL-TS	362.33a	50.50a	40.63b	95.00b	193.22a	35.33a	92.53a

Note: The letters in the same column indicate significant differences at the P <  0.05.

**Fig 3.**
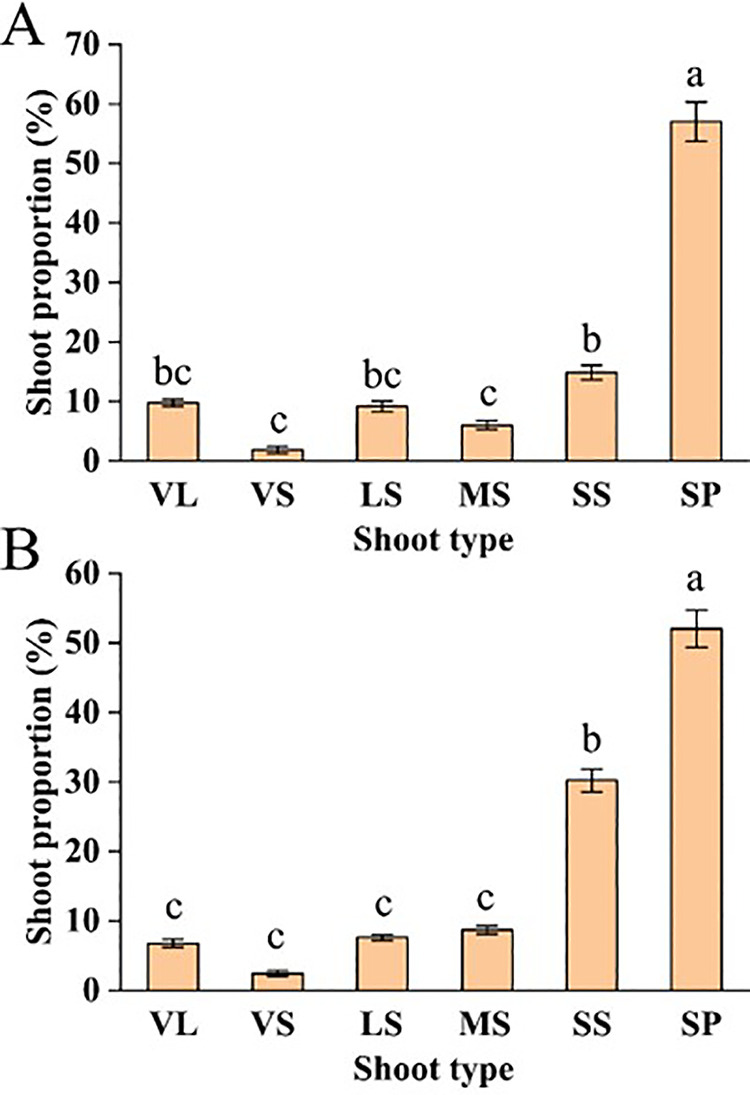
Proportion of SSS-TS and YLL-TS branches (A: SSS-TS(B:YLL-TS). [Note: Different small letters indicate significant difference at p<0.05, Abbreviation: LS, long fruit shoots (≥25cm); MS, middle fruit shoots (10-25 cm); SS, Short fruit shoots (5-10 cm); SP, spurs (≤2 cm); VL, vegetative long shoots (>30 cm); VS, vegetative short shoots (VS) (0-30 cm)].

As shown in Fig 3, the proportions of different branches between the two TSs varied. The proportion of bouquet-shaped fruiting branches (SP) was the highest, accounting for 53.81% in YLL-TS and 57.59% in SSS-TS. Among other branches, the proportion of medium-sized (MS) fruiting branches in YLL-TS was the lowest at 4.98%, while the proportion of vegetative-short (VS) branches in SSS-TS was the lowest at only 2.43%.

### 3.2 Canopy branch and leaf anisotropic growth relationships

The allometric growth relationship between branch and leaf morphological structures is fundamental for the three-dimensional reconstruction of the canopy. From the study it was observed that the correlation coefficients for the allometric growth relationships between different branches and leaves varied (Tables 2 and 3). In YLL-TS, the minimum correlation coefficient for the allometric growth relationship between branches and leaves was observed in the functional relationship between the length of the nutritional long branch and the length of the leaf petiole, with r² =  0.206. The maximum correlation coefficient was found in the functional relationship between the length of the short fruit branch and the number of branches and leaves, with r² =  0.851. In SSS-TS, the minimum correlation coefficient for the allometric growth relationship between branches and leaves was found in the functional relationship between the length of the medium fruit branch and the length of the leaf petiole, with r² =  0.211. The maximum correlation coefficient was observed in the functional relationship between the length of the nutritional long branch and the area of branches and leaves, with r² =  0.827.

**Table 2:** Allometric relationship of SSS-TS branches and leaves.

Morphology equations	Parameters	Shoot type					
		Spurs (SP)	Short fruit shoot (SS)	Middle fruit shoot (MS)	Long fruit shoot (LS)	Vegetative short shoot(VS)	Vegetative long shoot(VL)
Branch length and number of branches and leaves	Sample quantity	15	15	15	15	15	15
LN = a(SL) + b	Slope a	1.007	0.534	0.505	0.289	0.279	0.289
	Intercept b	4.99	6.03	7.265	7.444	5.451	0.865
	Coefficient of Determination r^2^	0.551	0.728	0.623	0.615	0.594	0.712
Branch length and area of branches and leaves	Slope a	65.889	51.762	35.316	8.529	14.831	21.306
SLA = a(SL) + b	Intercept b	9.843	27.895	133.31	222.86	185.4	536.68
	Coefficient of Determination r^2^	0.694	0.82	0.506	0.637	0.704	0.827
Leaf length and petiole length	Sample quantity	446	446	446	446	446	446
PL = a(LL) + b	Slope a	0.171	0.307	0.207	0.149	0.129	0.369
	Intercept b	0.978	0.262	0.712	1.368	1.574	1.618
	Coefficient of Determination r^2^	0.546	0.591	0.211	0.214	0.339	0.814
Leaf area and leaf length × leaf length	Slope a	0.112	0.32	0.254	0.322	0.262	0.224
LA = a(LL²)	Coefficient of Determination r^2^	0.351	0.872	0.735	0.853	0.802	0.697
Leaf width and leaf length	Slope a	0.316	0.526	0.283	0.443	0.257	0.286
LW = a(LL)	Coefficient of Determination r^2^	0.613	0.76	0.279	0.623	0.469	0.399

Note: Allometric growth relationship between canopy branches and leaves is the basic data for canopy 3D model construction.

**Table 3:** Allometric relationship of YLL-TS branches and leaves.

Morphology equations	Parameters	Shoot type						
	Spurs (SP)	Short fruit shoot (SS)	Middle fruit shoot (MS)	Long fruit shoot (LS)	Vegetative short shoot(VS)		Vegetative long shoot(VL)	
Branch length and number of branches and leaves	Sample quantity	15	15	15	15	15		15
LN = a(SL) + b	Slope a	0.656	1.134	0.925	0.438	0.254		0.482
	Intercept b	5.958	3.286	0.134	3.75	5.776		14.557
	Coefficient of Determination r^2^	0.415	0.851	0.759	0.74	0.664		0.808
Branch length and area of branches and leaves	Slope a	36.694	27.168	15.068	10.763	22.808		6.001
SLA = a(SL) + b	Intercept b	115.61	181.63	160.15	288.93	206.93		524
	Coefficient of Determination r^2^	0.467	0.277	0.386	0.236	0.942		0.269
Leaf length and petiole length	Sample quantity	339	339	339	339	339		339
PL = a(LL) + b	Slope a	0.354	0.241	0.232	0.136	0.153		0.188
	Intercept b	0.172	0.936	1.107	2.077	1.857		1.632
	Coefficient of Determination r^2^	0.749	0.416	0.427	0.344	0.243		0.206
Leaf area and leaf length × leaf length	Slope a	0.293	0.299	0.295	0.269	0.349		0.323
LA = a(LL²)	Coefficient of Determination r^2^	0.829	0.875	0.867	0.837	0.782		0.32
Leaf width and leaf length	Slope a	0.362	0.348	0.37	0.301	0.361		2.875
LW = a(LL)	Coefficient of Determination r^2^	0.554	0.642	0.58	0.52	0.481		0.426

Note: Allometric growth relationship between canopy branches and leaves is the basic data for canopy 3D model construction.

### 3.3 Canopy leaf Euler angle distribution

During the digitalization process of tree canopy layers, collecting numerical values of leaf angle for different branches of different TSs can provide the distribution range of spatial angles for various branch types, serving as the basic data for the reconstruction of canopy three-dimensional models.

**Fig 4:**
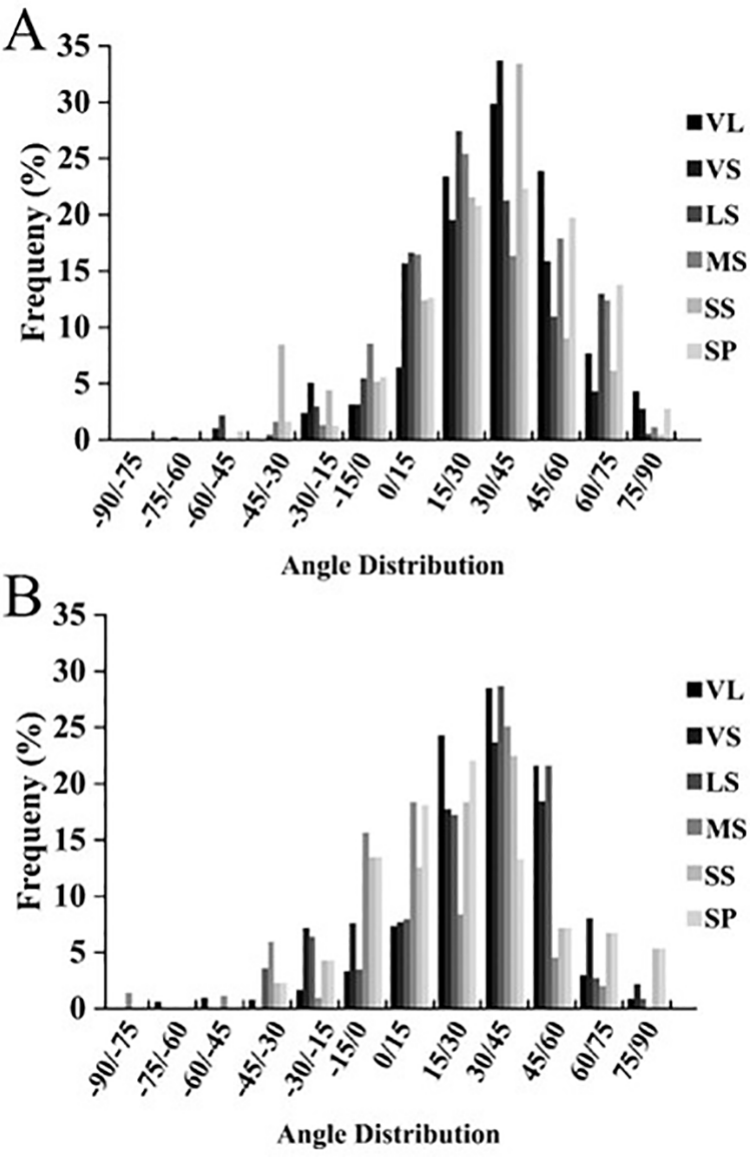
The leaf elevation angle distribution of different type shoots in SSS-TS (A) and YLL-TS (B). [Note: SP: Spurs; SS: Short fruit shoot; MS: Middle fruit shoot; LS: Long fruit shoot; VS: Vegetative short shoot; VL: Vegetative long shoot.].

As shown in Fig 4, there existed a similar distribution pattern of leaf height angles between different branch types of YLL-TS and SSS-TS. The leaf height angles of both TSs were primarily distributed within the range of (15°, 60°). The leaf height angles of nutrient long shoots and nutrient short shoots are mainly concentrated in the interval of (30°, 45°), with the proportion of nutrient long shoots and nutrient short shoots in YLL-TS being 30.91% and 25.73%, respectively, and in SSS-TS being 29.50% and 33.32%, respectively. Compared to SSS-TS, the highest proportions of leaf height angles of long fruiting branches, medium fruiting branches, and short fruiting branches of the YLL-TS were within the interval of (30°, 45°), accounting for 30.91%, 25.73%, 31.16%, 27.26%, and 24.38%, respectively. The leaf height angles of cluster-shaped fruiting branches were mainly distributed within the interval of (15°, 30°).

**Fig 5.**
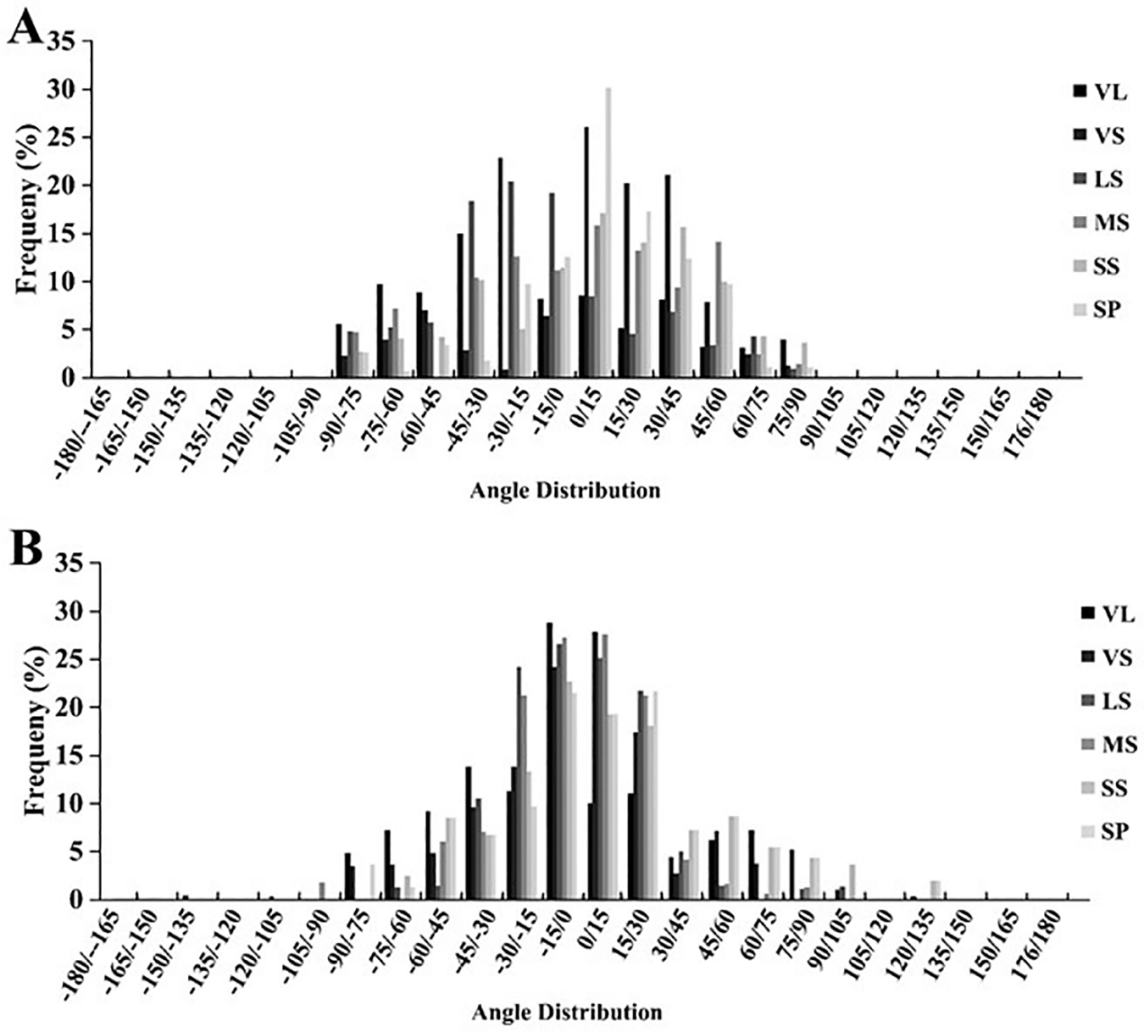
STAR values of various branch types in SSS-TS and YLL-TS. [Note: SP: Spurs; SS: Short fruit shoot; MS: Middle fruit shoot; LS: Long fruit shoot; VS: Vegetative short shoot; VL: Vegetative long shoot].

As shown in Fig 5, we found that the distribution range of leaf turning angles for various branch types of the YLL-TS was mainly within (-105°, 105°), which was wider than the distribution range of leaf turning angles for SSS-TS (-45°, 45°). The highest proportions of leaf turning angles for nutrient long shoots, long fruiting branches, short fruiting branches, and cluster-shaped fruiting branches of YLL-TS were within the interval of (-15°, 0°), accounting for 24.11%, 22.29%, 19.00%, and 18.00%, respectively. The leaf turning angles of nutrient short shoots are mainly distributed within the interval of (0°, 15°).

### 3.4 Canopy three-dimensional model construction and accuracy verification

**Fig 6:**
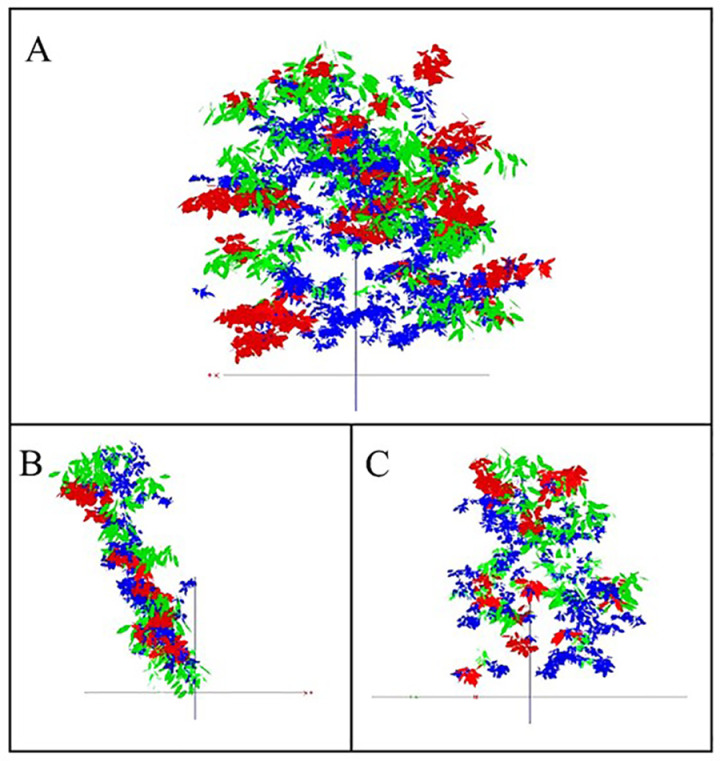
Virtual canopy images of SSS-TS (A) and YLL-TS (B) (C). [Note: Different colors represent different types of branches: (1) red: Vegetative long shoot and Vegetative short shoot; (2) green: Long fruit shoot and Middle fruit shoot; (3) blue: Short fruit shoot and Spurs. Images were synthesized with VegeSTAR4.0 software].

The virtual canopies for both TSs, obtained through digitization of the selected YLL-TS and the control SSS-TS (Fig 6). The simulated three-dimensional virtual canopies illustrated the spatial structures of the cherry trees with different shapes (Fig 6). They visually represent the spatial distribution of various branch types and leaves by employing different color schemes (Fig 6). YLL-TS canopy is inclined at a certain angle to the ground.

**Fig 7:**
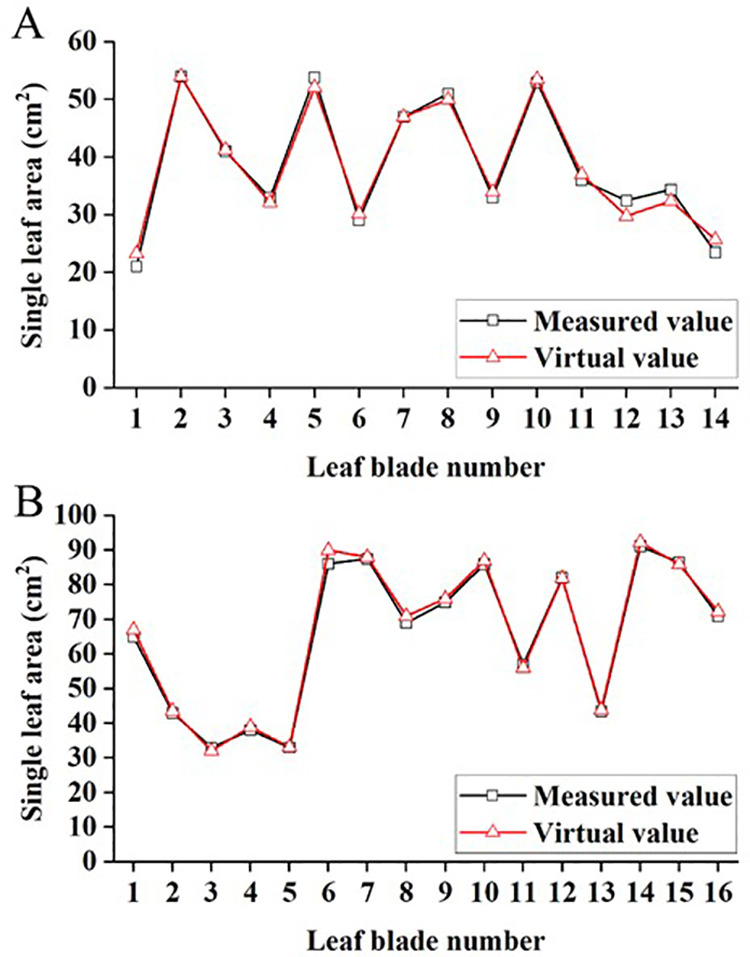
Comparison of virtual and actual leaf area (A:SSS-TS;B:YLL-TS).

Virtual canopy reconstruction can effectively showcase the spatial structure of tree canopies, but its simulation accuracy needs verification. To validate the accuracy of the three-dimensional simulated canopy, we compared simulated values of individual leaf area under the same branch length with measured values (Fig 7). There were differences in the correlation analysis results of the simulated values and measured values of individual leaf area for both TSs. Specifically, for YLL-TS, R² = 0.997, RMSE = 1.70 cm^2^, RE = 2.62%, while for SSS-TS, R² = 0.985, RMSE = 1.27 cm^2^, RE = 3.26%.

### 3.5 Analysis of canopy branching and foliage conditions

Based on the spatial coordinates of the tree canopy collected through digitization and the reconstructed three-dimensional models, we obtained detailed information about the branches and leaves of different TSs, including the number of branch types, branch lengths, and leaf numbers. The number of branches, total branch length, total number of branches and leaves, and leaf area of YLL-TS were all significantly lower than those of the control (Table 4). Specifically, YLL-TS had 333.33 branches, 34.76 m total branch length, 3223.19 total branches and leaves, and 13.91 m² leaf area. However, the average leaf area per branch was higher in YLL-TS than in the control, at 3013.07 cm²/branch.

**Table 4:** Comparison of branches and leaves of two TSs.

TS	Total shoot	Total shoot length (m)	Shoot leaf amounts	Total leaf area (m^2^)	Leaf area/shoot (cm^2^)
SSS-TS (CK)	684.67a	90.95a	7044.91a	21.41a	2710.12a
YLL-TS	333.33b	34.76b	3223.19b	13.91b	3013.07a

Note: The letters in the same column indicate significant differences at the P <  0.05.

### 3.6 Analysis of LIE in the canopy

As shown in Table 5, The LIE varied among different types of branches. YLL-TS consistently higher than the control. Additionally, significant differences were observed between cluster-shaped fruiting branches, long fruiting branches, and nutrient short shoots of YLL-TS compared to the control. The LIE of different types of branches in YLL-TS followed the order: medium fruiting branches =  nutrient short shoots>  cluster-shaped fruiting branches>  long fruiting branches>  nutrient long shoots>  short fruiting branches.

**Table 5:** STAR values of various types of SSS-TS and YLL-TS branches.

TS	Shoot type						
	Spurs (SP)	Short fruit shoot (SS)	Middle fruit shoot (MS)	Long fruit shoot (LS)	Vegetative short shoot(VS)	Vegetative long shoot (VL)	Total STAR
SSS-TS(CK)	0.20b	0.26a	0.50a	0.36b	0.43b	0.28a	0.29b
YLL-TS	0.53a	0.31a	0.59a	0.45a	0.59a	0.32a	0.43a

Note: STAR calculated by VegeSTAR4.0 on June 1, 2021; The letters in the same column indicate significant differences at the P <  0.05.

### 3.7 Comparison of photosynthetic characteristics of leaves of different TSs

Different TSs have varying impacts on the photosynthetic characteristics of sweet cherry leaves. As shown in Table 6, the values of transpiration rate (Tr), stomatal conductance (Gs), and intercellular CO_2_ concentration (Ci) differed among the different TSs and different parts of the same TS. Specifically, the net photosynthetic rate of leaves in the upper (2-3 m) and middle (1-2 m) canopy of SSS-TS was significantly higher than that of YLL-TS. However, there was no significant difference in the net photosynthetic rate of leaves in the lower (0-1 m) canopy between the two TSs. Moreover, the net photosynthetic rate of leaves in the middle canopy was higher than that in the upper and lower canopies for both TSs, with values of 14.66/μmol·m^-2^·s^-1^ and 14.13/μmol·m^-2^·s^-1^, respectively.

**Table 6:** Comparison of photosynthetic characteristics in different parts of two TSs.

Photosynthetic parameters	Top(2-3 m)		Middle(1-2 m)		Bottom(0-1 m)	
	SSS-TS	YLL-TS	SSS-TS	YLL-TS	SSS-TS	YLL-TS
net photosynthetic rate/μmol·m^-2^·s^-1^	13.03b	13.18a	14.66a	14.13b	12.26a	12.34a
Transpiration rate/mmol·m^-2^·s^-1^	9.53a	3.68b	8.66a	5.13b	7.51a	4.34b
Stomatal conductance/mol·m^-2^·s^-1^	0.54b	0.65a	0.36a	0.48a	0.52a	0.26b
Intercellular CO_2_ concentration/μmol·mol^-1^	317.27a	270.51b	341.75a	334.42b	254.43a	235.32b

Note: The letters in the same row indicate significant differences at the P <  0.05; The determination time was 9: 00-9: 30 am on May 15, 2021.

### 3.8 Comparison of fruit productivity and quality of different TSs

As shown in Table 7, SSS-TS exhibited the highest single fruit weight, soluble solids content, and fruit brightness, which were significantly higher than those of YLL-TS, with values of 11.05 g, 18.33%, and 32.736, respectively. There were not significant differences between the two TSs in terms of fruit hardness and the red-green (a*) and yellow-blue (b*) color values.

**Table 7:** Comparison of fruit quality of two TSs.

TS	Weight per fruit/g	Fruit firmness(g/mm^2^)	Soluble solid content/%	Brightness L *	Redness a *	Yellowish-blue degree b *
SSS-TS (CK)	11.05 ± 1.587a	242.89 ± 3.797a	18.33 ± 2.0087a	32.736a	30.405a	11.955a
YLL-TS	8.11 ± 0.776b	233.23 ± 3.844a	15.64 ± 0.8187b	30.931b	28.482a	9.810a

Note: Different letters in the same column indicate significant differences at the P <  0.05.

As shown in Table 8, we found that the different TSs, due to different planting densities, exhibited differences in both per plant and per acre yield, and all of them showed significant differences. Compared to the control, YLL-TS had fewer branches, resulting in a lower per plant yield of 22.70 kg/plant. However, the planting density per mu is higher than that of the control, leading to a significantly higher yield per mu of 4.075 kg/m^2^.

**Table 8:** Yield comparison of two TSs.

TS	Number of plants per 667m^2^	Yield per plant/kg	Yield per 667m^2^/kg
SSS-TS (CK)	74	31.94 ± 0.832a	2450.41 ± 1.012b
YLL-TS	126	22.70 ± 0.728b	2718.20 ± 0.918a

Note: Different letters in the same column indicate significant differences at the P <  0.05; Planting number per 667m^2^ and yield per 667m^2^ are calculated based on two cultivation patterns.

As shown in Table 9, YLL-TS had fewer branches per mu compared to the control. However, the yield per unit dry cross-sectional area and per unit leaf area were higher than those of the control. Furthermore, there was a significant difference in the yield per unit leaf area compared to the control, reaching 1.14 kg/m^2^.

**Table 9:** Comparison of orchard production efficiency with two TSs.

TS	Total Shoots (×10^4^/per 667m^2^)	Yield/TCSE (kg/cm^2^)	Yield/leaf area (Kg/m^2^)
SSS-TS(CK)	5.07a	0.13a	0.79b
YLL-TS	4.20a	0.18a	1.14a

Note: Different letters in the same column indicate significant differences at the P <  0.05.

## 4. Discussion

### 4.1 Feasibility of sweet cherry tree shape evaluation based on three-dimensional modeling

Reports on the structural characteristics of sweet cherry trees are relatively scarce, and quantitative analysis of canopy structure is limited. In this study, three-dimensional digitization technology was employed to reconstruct the canopy structures of two different tree shapes (TSs) of sweet cherry. The reconstructed models were then utilized to analyze branch and leaf distribution as well as the light interception efficiency (LIE) of the canopies.

The accuracy of virtual plant digitization is high, with the reconstruction of corresponding models based on tree structure relationships and biological principles [16]. Therefore, the obtained virtual plant models can realistically reflect tree structure. By digitizing the branches and leaves on-site, we accurately captured the spatial distribution patterns of different types of branches and leaf angles, reflecting the actual spatial structural characteristics of the trees. Using three-dimensional digitization technology, combined with algorithmic rules and growth parameters between branches and leaves, we constructed virtual canopy spatial structures of sweet cherry trees in both SSS-TS and YLL-TS. Error analysis indicated that the accuracy of the reconstructed models meets the requirements for analyzing the spatial canopy structure and light interception of sweet cherry trees. This technology enables the visualization, precision, and digitalization of sweet cherry tree canopy structures.

### 4.2 Effects of spatial angular distribution of leaves in different tree shapes

The spatial angle of sweet cherry tree leaves plays a crucial role in the LIE of the tree canopy, encompassing azimuthal, azimuth, and elevation angles [17]. Previous studies on leaf angles in various fruit trees have revealed differences in leaf angle distributions among different TSs, allowing trees to receive more sunlight and adapt to capture more solar energy [18]. The spatial angles of sweet cherry tree leaves, including azimuthal, azimuth, and elevation angles, are closely linked to the latitude where the trees are grown, as latitude determines the solar position and light availability throughout the year [19]. At lower latitudes, where sunlight is more direct and intense, leaves may orient themselves obliquely to optimize diffuse light capture and minimize the risk of photodamage caused by excessive radiation. Conversely, at higher latitudes Iin present experimentation), where sunlight arrives at lower angles and is less intense, leaves tend to adopt steeper angles to maximize interception of oblique sunlight, especially during the shorter growing seasons. These adjustments are critical for maintaining canopy Light Interception Efficiency (LIE) across varying solar radiation conditions [20]. Moreover, latitude influences seasonal changes in photoperiod and solar elevation, requiring canopy structures to adapt dynamically for optimal light capture. Such spatial leaf orientation strategies are crucial for photosynthesis and growth, particularly in species like sweet cherry, which are sensitive to light conditions [21].

Earlier reports on leaf spatial angles employed canopy analyzers with predetermined hypothetical values to reflect actual leaf angles, which may not accurately depict real distributions. In this study, we used three-dimensional digitization technology to directly collect spatial angles of leaves from different TSs of sweet cherry trees and conducted comparative analyses.

Our results indicated differences in azimuthal and azimuth angles between ‘Jimei’ SSS-TS and YLL-TS. Specifically, the elevation angles of leaves from different types of branches in both TSs mainly fell within the range of 15° to 60°. The elevation angle reflects the angle of leaves in the vertical direction on branches. There were no significant differences in the distribution of elevation angles of leaves across different canopy structures of the same sweet cherry variety. The similar distribution range of elevation angles in different TSs might be attributed to the relatively large leaf area of sweet cherry trees compared to other fruits like apples and peaches, and the large volume of the tree canopy, which may not necessitate larger elevation angles for receiving sunlight. Differences were observed in leaf azimuth angles between the TSs. The azimuth angles of leaves in SSS-TS were primarily distributed within -45° to 45°, while those in YLL-TS were mainly distributed within -105° to 105°. The wider range of leaf azimuth angles in YLL-TS may be attributed to its unique canopy structure, which allows leaves to receive light from a broader spatial range compared to SSS-TS.

### 4.3 Effect of canopy structure and lie of trees with different tree shapes

The light interception capacity of the canopy significantly affects the accumulation of dry matter in trees and fruit yield [22]. Pruning for fruit tree shaping must ensure a reasonable distribution of branch and leaf area and an adequate number of branches and leaves to ensure strong light interception capacity, enabling high-quality and high-yield fruit production [23]. Sweet cherries are photophilic, and sufficient and proper light distribution is crucial for their yield and fruit quality. In this study, we evaluated the light interception capacity and canopy structure of two different shapes of sweet cherry trees using three-dimensional digitization technology.

The precise description of canopy structure using virtual models of sweet cherry trees allows accurate depiction of canopy structure [24]. We evaluated the canopy LIE of different TSs by simulating the light environment and calculating the ratio of projected leaf area (PLA) to total leaf area (TLA), known as the STAR value. The STAR value describes the effective leaf area for capturing light in the canopy. Comparing the canopy LIE of the two different shapes of sweet cherry trees, we found that YLL-TS exhibited significantly higher LIE than SSS-TS. YLL-TS, with its trellis-like canopy structure, retained only the main branches in the north-south direction during pruning, keeping the east-west growing branches at a low rate to ensure the canopy captures sunlight and addresses the problem of canopy closure in sweet cherries. Although the SSS-TS had more branches, leaf area, and total leaf numbers compared to YLL-TS, its LIE was significantly lower, possibly due to mutual shading effects among branches and leaves.

There were significant differences in the light interception capacity of the two different shapes of sweet cherry trees, likely related to canopy structure. According to Table 4, the SSS-TS had more branches, resulting in higher total leaf area and leaf number compared to the YLL-TS, leading to significantly lower LIE due to mutual shading between branches and leaves. While the YLL-TS exhibited higher canopy LIE than the SSS-TS, its fruit yield was limited and significantly lower than that of the latter. The yield per tree of SSS-TS was significantly higher due to a greater quantity of bouquet-like fruiting branches.

Table 4 shows that there was no significant difference in the average leaf area of different types of branches between different TSs. The average leaf area of the SSS-TS’s nutritional long branches was 720.35 cm²/branch, while that of the YLL-TS was 904.83 cm²/branch. No significant differences were observed in the average leaf area of other types of branches, indicating no significant difference in the average leaf area of branches between different shapes of the same sweet cherry variety. According to Tables 4 and 5, although the bouquet-like fruiting branches of the SSS-TS had higher leaf area, their LIE was lower than that of other branch types, possibly due to their distribution within the inner canopy, which is not conducive to light interception. In comparison, the LIE of bouquet-like fruiting branches of the YLL-TS was significantly higher. The predominantly north-south growth direction of branches in the YLL-TS is more conducive to capturing light energy, resulting in significantly higher LIE overall.

### 4.4 Effect of different tree shapes on fruit yield

Research suggests that the LIE of fruit tree canopies greatly benefits tree growth and the quality and quantity of fruit production [22]. The SSS-TS of sweet cherries has a lower planting density per acre compared to the YLL-TS (Table 8), but its yield per tree is significantly higher. Additionally, due to the higher planting density of the YLL-TS, even though the yield per tree is lower than that of the SSS-TS, its yield per acre is significantly higher. According to Table 9, the yield per unit leaf area of the YLL-TS reaches 1.14 kg, whereas the SSS-TS only achieves 0.79 kg. The YLL-TS not only improves land utilization but also offers better ventilation and light transmission with fewer branches and leaf area, making it suitable for the current trend of dwarf and dense planting.

## 5. Conclusions

The study utilized three-dimensional digitization technology to digitize the canopies of two different shapes of sweet cherry trees and used virtual plant technology to reconstruct the canopy structure, evaluate the canopy’s LIE, and compare the fruit yield of the two shapes. The YLL-TS has a better LIE compared to the SSS-TS and has a higher planting density per acre (126 plants/ 667 m^2^), despite fruit produced from SSS-TS has better quality attributes. To produce fruits with a single fruit weight of over 8g per kg, only 0.79 m² of leaf area is required, which is more efficient than the 1.14 m² required for the SSS-TS. Therefore, adopting the YLL-TS is more suitable for the trend of dwarf and dense cultivation of sweet cherries.
